# Neoadjuvant plus adjuvant combined or sequenced vemurafenib, cobimetinib and atezolizumab in patients with high-risk, resectable BRAF-mutated and wild-type melanoma: NEO-TIM, a phase II randomized non-comparative study

**DOI:** 10.3389/fonc.2023.1107307

**Published:** 2023-02-09

**Authors:** Paolo A. Ascierto, Eleonora Cioli, Vanna Chiarion-Sileni, Pietro Quaglino, Francesco Spagnolo, Massimo Guidoboni, Michele Del Vecchio, Ketty Peris, Paola Queirolo, Luisa Fioretto, Corrado Caracò, Miriam Paone, Antonio Sorrentino, Mariaelena Capone, Diana Giannarelli, Gerardo Ferrara, Daniela Massi, Claudia Trojaniello

**Affiliations:** ^1^ Department of Melanoma, Cancer Immunotherapy and Development Therapeutics. Istituto Nazionale Tumori IRCCS Fondazione “G. Pascale” Napoli, Naples, Italy; ^2^ Melanoma Oncology Unit, Veneto Institute of Oncology IOV-IRCCS, Padova, Italy; ^3^ Department of Medical Sciences, Dermatologic Clinic, University of Turin, Turin, Italy; ^4^ Skin Cancer Unit, IRCCS Ospedale Policlinico San Martino, Genova, Italy; ^5^ Immunotherapy and Cell Therapy Unit, Istituto Scientifico Romagnolo per lo Studio e la Cura dei Tumori (IRST) IRCCS, Meldola, Italy; ^6^ Unit of Melanoma Medical Oncology, Department of Medical Oncology and Hematology, Fondazione IRCCS Istituto Nazionale dei Tumori, Milan, Italy; ^7^ Catholic University of the Sacred Heart and Fondazione Policlinico Universitario A. Gemelli-IRCCS, Rome, Italy; ^8^ Division of Melanoma Sarcoma and Rare Tumors, IEO European Institute of Oncology IRCCS Milan, Milan, Italy; ^9^ Medical Oncology Unit, Department of Oncology, Santa Maria Annunziata Hospital, Azienda USL Toscana Centro, Florence, Italy; ^10^ Melanoma and Skin Cancers Surgery Unit, Istituto Nazionale Tumori IRCCS Fondazione “G. Pascale”, Napoli, Italy; ^11^ Fondazione Policlinico Universitario A. Gemelli, IRCCS – Facility of Epidemiology & Biostatistics, Rome, Italy; ^12^ Department of Pathology and Cytopathology, Istituto Nazionale Tumori IRCCS Fondazione “G. Pascale”, Napoli, Italy; ^13^ Section of Pathology, Department of Health Sciences, University of Florence, Florence, Italy

**Keywords:** metastatic melanoma, cobimetinib, vemurafenib, atezolizumab, neoadjuvant therapy

## Abstract

**Background:**

Following the increased survival of patients with metastatic melanoma thanks to immunotherapy and targeted therapy, neoadjuvant approaches are being investigated to address the unmet needs of unresponsive and intolerant patients. We aim to investigate the efficacy of neoadjuvant plus adjuvant combined or sequenced vemurafenib, cobimetinib and atezolizumab in patients with high-risk, resectable *BRAF*-mutated and wild-type melanoma.

**Methods:**

The study is a phase II, open-label, randomized non-comparative trial in patients with stage IIIB/C/D surgically resectable, *BRAF*-mutated and wild-type melanoma, with three possible treatments: (1) vemurafenib 960 mg twice daily from day 1 to 42; (2) vemurafenib 720 mg twice daily from day 1 to 42; (3) cobimetinib 60 mg once daily from day 1 to 21 and from day 29 to 42; and (4) atezolizumab 840 mg for two cycles (day 22 and day 43).

Patients will be randomized to three different arms: A) *BRAF*-mutated patients will receive over 6 weeks (1) + (3); B) *BRAF*-mutated patients will receive over 6 weeks (2) + (3) + (4); C) *BRAF* wild-type patients will receive over 6 weeks (3) + (4). All patients will also receive atezolizumab 1200 mg every 3 weeks for 17 cycles after surgery and after a second screening period (up to 6 weeks).

**Discussion:**

Neoadjuvant therapy for regional metastases may improve operability and outcomes and facilitate the identification of biomarkers that can guide further lines of treatment. Patients with clinical stage III melanoma may especially benefit from neoadjuvant treatment, as the outcomes of surgery alone are very poor. It is expected that the combination of neoadjuvant and adjuvant treatment may reduce the incidence of relapse and improve survival.

**Clinical trial registration:**

eudract.ema.europa.eu/protocol.htm, identifier 2018-004841-17.

## 1 Introduction

The survival of patients with stage IV melanoma has recently improved due to anti-PD-1 immunotherapies and BRAF/MEK combination targeted therapies, up to the recently reported 5-year overall survival (OS) rate of 52% with the combination ipilimumab/nivolumab (1–5). Almost 50% of patients with metastatic melanoma have the *BRAF*V600 mutation, with features of oncogene addiction to the *BRAF*-mutated gene (6). Translational data suggest that MEK and BRAF inhibition of oncogenic MAPK signaling can potentiate host antitumor immune response through its effects on T cells, including upregulation of melanoma antigen expression, upregulation of programmed death-ligand 1 (PD-L1) expression, and increased tumor T-cell infiltration (7–11). Combinations of MEK inhibitors with anti-PD-1/PD-L1 therapy have demonstrated synergistic tumor growth inhibition and reactivation of antitumor immunity in murine models (12). Moreover, early-phase clinical studies have demonstrated promising antitumor activity and restoration of antitumor immunity with combinations of MEK inhibitors and anti-PD-L1 antibodies in patients with BRAFV600 wild-type advanced melanoma (13).

In a phase I study (NCT01988896), the combination of cobimetinib, a MEK inhibitor, with atezolizumab, an anti-PD-L1 antibody, had promising anti-tumor activity. Indeed, the CD8 + T-cell infiltration and the MHC I expression in tumor tissue increased after starting the cobimetinib run-in and adding atezolizumab to cobimetinib compared to baseline (14). Contrary to these results, the phase III trial Imspire170, which compared pembrolizumab alone with atezolizumab and cobimetinib combination in patients with *BRAF*V600 wild-type (WT) advanced melanoma, did not meet the primary endpoint (15). This study showed a higher benefit with the combination than with anti-PD-1 alone in the first 4 months, suggesting the use of short-course MEK inhibitor + anti-PD-1/PD-L1 in a neoadjuvant setting (15, 16).

In addition, treatment with atezolizumab and vemurafenib (a BRAF serine–threonine kinase inhibitor) with or without cobimetinib had higher response rates and faster and longer-lasting responses than cobimetinib plus vemurafenib or atezolizumab alone in previously untreated *BRAF*V600 mutation-positive advanced melanoma (17). The ongoing IMspire 150 phase III trial compares the combination cobimetinib-vemurafenib to either atezolizumab or placebo in *BRAF*v600 mutation-positive unresectable or advanced melanoma (18). At a median follow-up of 18.9 months (IQR: 10.4–23.8), the investigator-assessed progression-free survival was significantly longer with atezolizumab compared with placebo (15.1 vs 10.6 months; hazard ratio [HR]: 0.78; 95% CI: 0.63–0.97; p=0.025). Moreover, the median duration of response was longer in the atezolizumab group (21.0 months; 95% CI: 15.1–not estimable) than in the control group (12.6 months; 95% CI: 10.5–16.6), while safety was comparable in the two groups (18).

To address unmet needs for unresponsive and intolerant patients, neoadjuvant approaches arebeing investigated. This approach could provide blood and tumor tissue samples, obtained before and after the systemic therapy, for biomarker assessment and better treatment individualization Moreover, neoadjuvant treatment may avoid extensive unnecessary surgery increasing HRQOL (19), and could increase the proportion of RO resections (20).

It has recently been demonstrated that treatment with neoadjuvant and adjuvant targeted therapy with dabrafenib and trametinib is associated with a high pathologic complete response (pCR) rate and improved outcomes over surgery alone (21). Also, treatment with immune checkpoint blockade (ICB) studied in the neoadjuvant setting has provided very high pathologic rates and low recurrence rates in responding patients. Moreover, preclinical studies suggest that neoadjuvant administration of ICB is associated with improved survival and enhanced and long-lasting anti-tumor immune response compared with the same therapy administered in the adjuvant setting (22).

Prospective neoadjuvant clinical trials with targeted (dabrafenib/trametinib combination) or immunotherapeutic agents (nivolumab alone or nivolumab/ipilimumab combination) are ongoing in high-risk melanoma patients with overall promising preliminary results (20, 21, 23). In a pooled analysis of six clinical trials of anti-PD-1-based immunotherapy or BRAF/MEK-targeted therapy, a pCR occurred in 47% of patients with targeted therapy and 33% with immunotherapy (43% combination and 20% monotherapy). Accordingly, pCR correlated with improved recurrence-free survival (2-year pCR 89% versus no pCR 50%, p<0.001) and OS (2-year pCR OS 95% versus no pCR 83%, p=0.027) (24).

Based on current evidence, we planned to investigate neoadjuvant with adjuvant treatment with targeted therapy and immunotherapy in combination or sequence recording anti-tumor activity risk and site of relapse after surgery, safety, and correlation between clinical and pathologic responses in high-risk surgically resectable, melanoma (stage III B/C/D).

## 2 Methods and analysis

### 2.1 Study design

**Figure 1:**
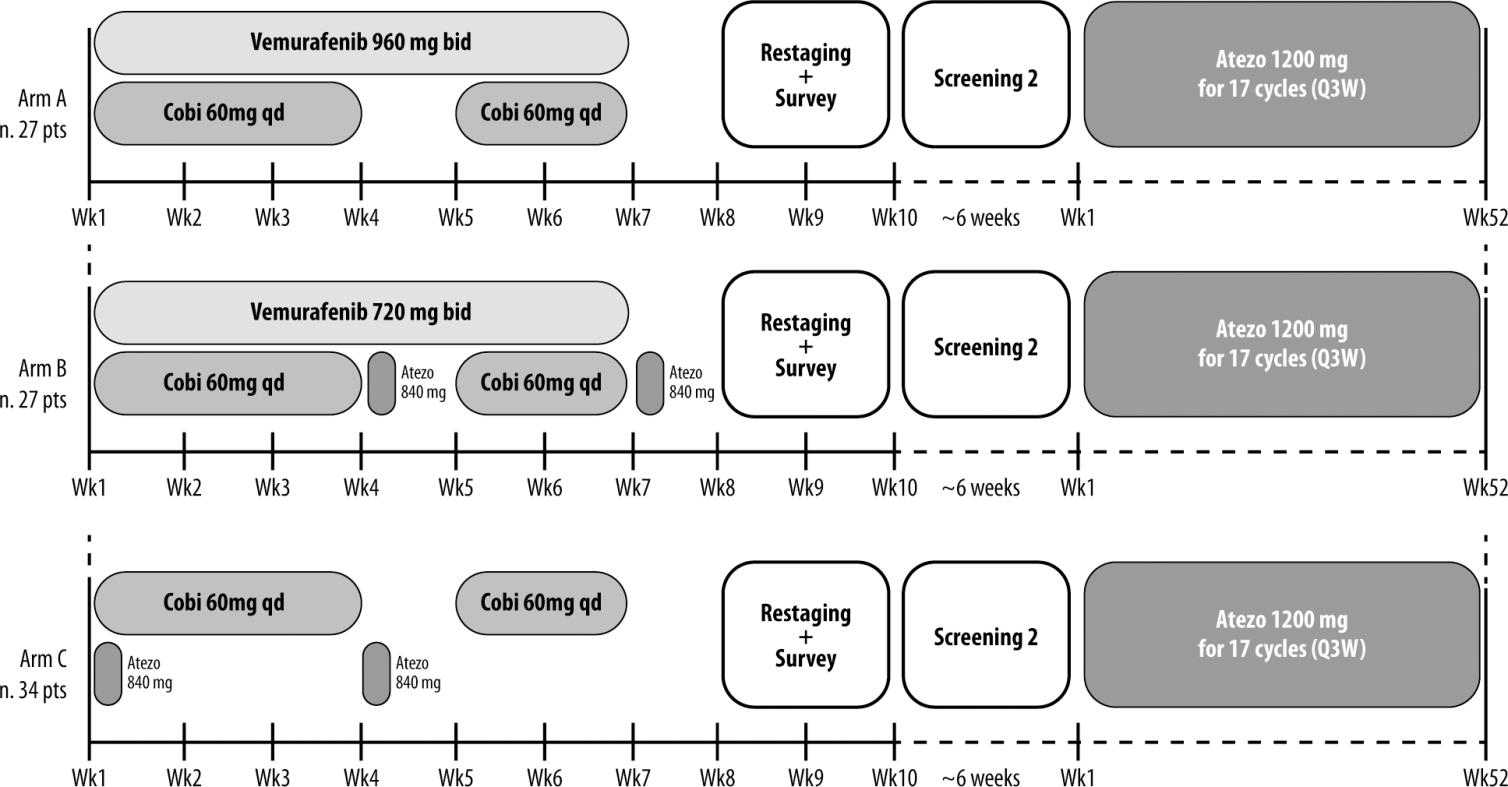
Study design.

NEO-TIM is a phase II, open-label, randomized non-comparative trial with neoadjuvant plus adjuvant therapy in combination or sequence, of targeted therapy and immunotherapy, in patients with stage IIIB/C/D surgically resectable, *BRAF*-mutated and WT melanoma. The study design is presented in **Figure 1**.

### 2.2 Selection of subjects

The study will enroll 88 subjects with surgically resectable *BRAF*-mutated and *BRAF* WT cutaneous melanoma. The detailed inclusion and exclusion criteria are summarized in **Table 1**.

Table 1Eligibility criteria for NEOTIM.

**Table:** 

Key inclusion criteria
•Patients of either sex aged ≥18 years Q11 •Capable of giving written informed consent, which includes compliance with the requirements and restrictions listed in the consent form •Patients must have histologically or cytologically confirmed stage IIIB/C/D 1 resectable cutaneous melanoma O The definition of resectability can be determined by the patient’s surgical oncologist and verified via discussion at the team of the Multidisciplinary Tumor Conference attended by melanoma medical and surgical oncology staff. Resectable tumors have no significant vascular, neural, or bony involvement. Only cases where a complete surgical resection with tumor-free margins can safely be achieved are defined as resectable •All patients must have a BRAF V600E/K mutation status known •Patients must be medically fit enough to undergo surgery as determined by the surgical oncology team •Patients must have measurable disease, defined by RECIST 1.1 •ECOG performance status 0–1 •Patients must have adequate organ and marrow function

**Table:** 

Key exclusion criteria
•No previous cancer therapy (chemotherapy, radiation therapy, immunotherapy, or biologic therapy) •Prior malignancy except for the following: adequately treated basal cell or squamous cell skin cancer, in situ cervical cancer, thyroid cancer (except anaplastic), or any cancer from which the patient has been disease-free for 2 years •Any major surgery within the last 3 weeks •Uncontrolled diabetes, hypertension, or other medical conditions that may interfere with the assessment of toxicity •Current use of anticoagulants (warfarin, heparin, direct thrombin inhibitors) at therapeutic levels •History of uncontrolled cardiovascular or interstitial lung disease and evidence or risk of retinal vein occlusion or central serous retinopathy •Subjects with conditions requiring systemic treatment with corticosteroids (>10 mg daily prednisone equivalents) or other immunosuppressive medications within 14 days of treatment. •Prior BRAF- or MEK-targeted therapy; patients who have received prior interferon are eligible •History of retinopathy or any finding at the ophthalmologic examination that is considered a risk factor for neurosensory retinal detachment/central serous chorioretinopathy, retinal vein occlusion, or neovascular macular degeneration •Prior BRAF- or MEK-targeted therapy; patients who have received prior interferon are eligible. •History of ocular/uveal/mucosal melanoma •Presence of any of the following risk factors for retinal vein occlusion: O Uncontrolled glaucoma with intra-ocular pressures ≥21 mmHg O Serum cholesterol grade ≥2; O Hypertriglyceridemia grade ≥2 O Hyperglycemia (fasting) grade ≥2 •Correct QT interval >450 ms to baseline, history of congenital long QT syndrome •Uncontrolled medical conditions, among which endocrine disorders (such as hypothyroidism, hyperthyroidism, and diabetes) •Other severe medical or psychiatric conditions (e.g., depression) or abnormalities of laboratory tests that may increase the risk associated with study participation or the assumption of vemurafenib, atezolizumab, and cobimetinib or that may interfere with the interpretation of study results, which in the judgment of the Investigator can make the patient not eligible for the study •Uncontrolled intercurrent illness, including but not limited to: ongoing or active infection, symptomatic congestive heart failure, uncontrolled hypertension, unstable angina pectoris, cardiac arrhythmia, cerebrovascular accident or transient ischemic attack, pulmonary embolism, interstitial lung disease, chronic gastrointestinal serious conditions associated with diarrhea or psychiatric illness/social situations that would limit compliance with study requirement, substantially increase risk of incurring adverse events or compromise the ability of the patient to give written informed consent •History of active primary immunodeficiency •Receipt of live attenuated vaccine within 30 days before the first dose. Note: enrolled patients should not receive a live vaccine while receiving study treatments and up to 30 days after the last dose of study treatment •Prior treatment with an anti-PD-1, anti-PD-L1, anti-PD-L2, or anti-CTLA-4 antibody •Known allergy or hypersensitivity to any of the study drugs or any of the study drug excipients •Positive test for HBV sAg or hepatitis C virus ribonucleic acid (hepatitis C virus antibody) indicating acute or chronic infection •Known history of testing positive for HIV or known AIDS •Judgment by the investigator that the patient is unsuitable to participate in the study and the patient is unlikely to comply with study procedures, restrictions, and requirements.

### 2.3 Treatments

Cobimetinib (provided by Hoffmann-La Roche) is an oral selective inhibitor of the MEK pathway. Vemurafenib (provided by Hoffmann-La Roche) is a low-molecular-weight inhibitor of BRAF serine–threonine kinase. Atezolizumab (provided by Hoffmann-La Roche) is an anti-PD-L1 antibody.

The study will include three possible treatments: (1) vemurafenib 960 mg twice daily per os from day 1 to 42; (2) vemurafenib 720 mg twice daily per os from day 1 to 42; (3) cobimetinib 60 mg once daily per os from day 1 to 21 and from day 29 to 42; cobimetinib should not be taken on days 22–28; and (4) atezolizumab 840 mg intravenous for two cycles (days 22 and 43).

Patients will be randomized to three different arms: A) *BRAF*-mutated patients will receive (1) + (3) for 6 weeks; B) *BRAF*-mutated patients will receive (2) + (3) + (4) for 6 weeks; C) *BRAF* WT patients will receive (3) + (4) for 6 weeks.

All patients will also receive atezolizumab 1200 mg intravenous every 3 weeks for 17 cycles after surgery and after a second screening period (up to 6 weeks).

### 2.4 Endpoints and assessments

Endpoints are reported in **Table 2**. The primary endpoint will be the pCR, defined as residual cancer burden = 0.

**Table 2:** Study endpoints.

Primary endpoint	
•To determine the pCR rate
Secondary endpoints
•Recurrence-free survival at 2 years, 3 years, and at the end of the study. Recurrence-free survival is defined as the time from randomization to recurrence event (local or distant disease development or death). •Overall survival is defined as the time from randomization to death. •Pathological overall response rate is defined as the sum of pCR, near pCRs and pathologic partial responses. •Safety: frequency of the following treatment-emergent AEs while on treatment or within 30 days after the last study treatment: All AEs, grade 3–5 AEs, serious AEs, AEs of special interest, and AEs leading to treatment discontinuation or withdrawal from the study. •To determine molecular and immunophenotypic changes in tumor and peripheral blood by evaluating several biomarkers. Since identifying new markers for immunotherapy is rapidly evolving, the definitive list of analyses remains to be determined. The following tests are suggested (differences: baseline, during the adjuvant treatment at week 6, surgical time points, and at recurrence will be compared): •Immunoscore (densities of tumor-infiltrating CD3 and CD8 cells), as well as PD-L1 expression on tumor and immune cells, evaluated by immunohistochemical analysis with an automated quantification system and a standardized assay on tumor tissue •Circulating cytokines and chemokines profiling, evaluated by Luminex technology in patient peripheral blood samples •Myeloid-derived suppressors cells and immune cell subtypes expression and lymphocyte activation, evaluated by multicolor cytofluorimetric approach in patient peripheral blood samples •Metabolomic profiling in patient peripheral blood samples, evaluated by nuclear magnetic resonance Spectrometer (600 MHz) •Tumor mutational burden by whole-exome sequencing on tumor and matched normal tissue at baseline. •Additional analysis of protein levels (i.e., CCR5), DNA mutations, and/or mRNA analysis to enable molecular classification (e.g., CMS), as well as other exploratory markers related to immunotherapy in tumor tissues

AE: Adverse event; pCR: Pathologic complete response.

Secondary endpoints will include recurrence-free survival at 2 years, 3 years and at the end of the study, defined as the time from randomization to recurrence event (local or distant disease development or death); patients without events at the end of the study period, who will be censored at the date of the last follow-up. OS is defined as the time from randomization to death; for patients alive at the end of the study, time will be censored at the date of the last follow-up. Pathological overall response rate is defined as the sum of pCRs, near pCRs and pathologic partial responses. Frequency of treatment-emergent adverse events (AEs) will be assessed during treatment or within 30 days after the last study treatment (all AEs, grade 3–5 AEs, serious AEs, AEs of special interest, and AEs leading to treatment discontinuation or withdrawal from the study). Molecular and immunophenotypic changes in the tumor and the peripheral blood will be evaluated by assessing biomarkers at baseline, during the adjuvant treatment at week 12 surgical time points, and recurrence. The list of markers will be completed based on updated evidence. At the time this protocol is prepared, the following biomarkers are envisaged: Immunoscore (densities of tumor-infiltrating CD3 and CD8 cells) and PD-L1 expression on tumor and immune cells, evaluated by immunohistochemical analysis with an automated quantification system and a standardized assay on tumor tissue; circulating cytokines and chemokines profiling, evaluated by the Multiplex Luminex technology (Luminex, Austin, TX, USA) in peripheral blood samples; myeloid-derived suppressor cells and immune cell subtypes expression and lymphocyte activation, evaluated by a multicolor cytofluorimetric approach in patient peripheral blood samples; metabolomic profiling in patient peripheral blood samples, evaluated by 1H-NMR (proton nuclear magnetic resonance) using a Bruker Avance 600 NMR spectrometer operated at a 599.97 MH; tumor mutational burden by whole-exome sequencing on tumor and matched normal tissue at baseline. Additional analysis of protein levels (i.e., CCR5), DNA mutations, and/or mRNA analysis is planned to enable molecular classification (e.g., CMS) and other exploratory markers related to immunotherapy in tumor tissues.

Some biomarkers and parameters proposed as potential predictive biomarkers for immunotherapy (i.e., C-reactive protein; LDH; absolute neutrophil, monocyte, eosinophil, and lymphocyte counts) will be correlated with pathological response and patient outcome.

### 2.5 Sample size

This study is designed as a phase II randomized, non-comparative trial, with pCR as the primary endpoint. According to Amaria et al. (21), a 60% pCR rate may be assumed in arms A and B. A sample size of 27 patients in each arm is sufficient to give at least a 90% probability of rejecting a 30% pCR rate with an exact 5% one-sided significance test when the true pCR rate is 60%. The null hypothesis of a pCR of 30% will be rejected if a pCR is observed in at least 12 patients.

According to Huang et al. (25), a pCR rate of about 30% may be assumed in arm C. A sample size of 34 patients is sufficient to give an 80% probability of rejecting a pCR rate of 15% with an exact 5% one-sided significance test when the true pCR rate is 35%. The null hypothesis of a pCR of 10% will be rejected if a pCR is observed in at least 10 patients.

### 2.6 Data analysis

All enrolled patients receiving at least one dose of the study medication will be considered the intention-to-treat population (ITT). The patient subgroup of the ITT population receiving at least one dose of the study drug will define the safety population. The analysis of efficacy endpoints will be performed in the ITT population, whereas the safety analysis will be performed in the safety population. A primary data analysis assessing the primary endpoint will be available after the neoadjuvant phase. A final data analysis will be available at study closure. The safety analysis will be completed within 12 months from the last visit of the last patient.

Primary endpoint results will be reported as counts and rates with confidence intervals. No comparisons among arms are planned. Demographical and clinical characteristics will be presented as descriptive statistics and summarized according to the treatment arm. Categorical variables will be given as counts and percentages, quantitative ones as mean and standard deviation, or median and interquartile range, as appropriate. No adjustment for multiple testing will be made.

Safety and tolerability will be assessed regarding AEs, laboratory data, ECG data, vital signs, and weight, which will be collected for all patients. AEs [both in terms of MedDRA preferred terms (26) and CTCAE 5.0 grade (27)], laboratory data, ECG data, vital signs data, and weight will be listed individually by the patient and summarized. Prevalence of related AEs, by grade, will be presented with a 95% CI. Survival will be represented with Kaplan-Meier curves, from which the median with 95% CIs will be derived.

Due to the small sample size, statistical analysis of biomarkers data will be conducted with the aim of hypothesis generation. A complete description of the data will be done. For biomarkers that might change over time due to treatment, levels before and after treatment will be compared with appropriate statistical tests based on the data type. Correlation with outcomes will be evaluated with univariate regression models. p ≤ 0.05 will be considered significant, and no adjustment is planned for multiple comparisons due to the exploratory nature of the analysis.

## 3 Discussion

Although recent advances in the treatment of metastatic melanoma have improved outcomes and survival, resistance and toxicity continue to be responsible for disease progression and mortality.

Neoadjuvant therapy for regional metastases has an established role in several cancers and is investigational in advanced melanoma (24). It may provide advantages such as improving operability and outcomes and facilitating the identification of biomarkers that can guide further lines of treatment.

Patients with clinical stage III melanoma may especially benefit from neoadjuvant treatment because outcomes of surgery alone are very poor. Two phase 2 open-label trials, one with neoadjuvant dabrafenib and trametinib and one with neoadjuvant talimogene laherparepvec, compared to upfront surgery, in advanced resectable melanoma, obtained meaningful benefit with increased event free survival (21, 28).

Triple therapy in the first line obtained encouraging results in ongoing phase III trials in advanced resectable melanoma. Pembrolizumab + dabrafenib + trametinib improved PFS, duration of response (DOR), and OS compared with placebo + dabrafenib + trametinib (29), and atezolizumab + vemurafenib + cobimetinib prolonged PFS and provided a clinically meaningful benefit in median DOR compared with placebo + vemurafenib + cobimetinib (30) in patients with *BRAF*V600E/K-mutant melanoma. Based on such results, this study will evaluate the neoadjuvant treatment with ICB and targeted therapy followed by adjuvant treatment with atezolizumab (an anti-PD-L1). It is expected a good number of pathological responses and a reduction of relapse incidence as well as a survival increase. This study may also help identify biomarkers and understand response mechanisms, analyzing tissue and blood samples obtained before and after systemic treatment.

This study will explore the concomitant or sequence administration of ICB and BRAF inhibitor/MEK/inhibitor, using an FDA approved triple-drug combination, vemurafenib/cobimetinib/atezolizumab. The data from such study will be comparable with data from the pooled analysis (24) and with the recent NeoTrio study (31). NeoTrio found that concurrent dabrafenib/trametinib/pembrolizumab had higher pCR and pathological response rate compared with dabrafenib/trametinib with sequence pembrolizumab and with pembrolizumab alone, but it had a high toxicity (31). Our study, differently from NeoTrio study, will use the triple combination with vemurafenib/cobimetinib/atezolizumab which gave positive results in the phase III study in patients with metastatic melanoma (18, 30) and is currently approved by FDA. Data from the *BRAF* wild-type arm of our study will also be useful to understand whether MEK inhibitors have immune-stimulating or immune-suppressor effect in advanced melanoma.

Comparison of trials may add useful information for the knowledge of this complex therapeutic strategy. As an example, it is known that the pooled analysis by Menzies et al. (24) of six trials on neoadjuvant therapy showed that not only pCR but also partial responses to neoadjuvant treatment gave a clinical benefit. However, this result is not in agreement with the newer PRADO study (phase II study of personalized response-directed surgery and adjuvant therapy after neoadjuvant ipilimumab and nivolumab in clinical stage III melanoma), which confirmed that patients with major pathologic response to neoadjuvant, without adjuvant therapy, had high relapse-free survival and distant metastasis free survival rates. Nevertheless, it was found that patients with partial pathologic response had worse outcomes, with no benefit for 2-year recurrence rate, and should receive adjuvant treatment (32). Therefore, previous data still need confirmation.

In conclusion, we planned a phase II randomized non-comparative study to evaluate neoadjuvant plus adjuvant combined or sequenced vemurafenib, cobimetinib and atezolizumab in patients with high-risk, resectable *BRAF*-mutated and wild-type melanoma, and we expect to improve outcomes in patients with advanced melanoma, with a better insight on the concerned populations through the pathologic examination of tissue samples.

## Ethics

This clinical study was designed, shall be implemented, and will be reported in accordance with the ICH Harmonized Tripartite Guidelines for Good Clinical Practice, with applicable local regulations (including European Directive 2001/20/EC), and with the ethical principles of the Declaration of Helsinki. This protocol, the Informed Consent Forms, any information to be given to the patient, and relevant supporting data have been approved by the Ethics Committee of the Principal Investigator (National Tumor Institute, “G. Pascale” Foundation, U.O.C. Melanoma, Oncology Immunotherapy, and Novel Therapies, Naples, Italy) and will be reviewed before the study is initiated. The study is registered at eudract.ema.europa.eu/protocol.htm (number 2018-004841-17). The key design elements of this protocol will be posted in the publicly accessible database clinicaltrials.gov: NCT04722575. Patient recruitment will be performed at Istituto Nazionale dei Tumori, Fondazione “G. Pascale”, Naples, Italy.

## Data availability statement

The original contributions presented in the study are included in the article/supplementary material. Further inquiries can be directed to the corresponding author.

## Ethics statement

The studies involving human participants were reviewed and approved by Institutional Review Boards of Istituto Nazionale Tumori IRCCS–Fondazione “G Pascale”. The patients/participants provided their written informed consent to participate in this study.

## Author contributions

All authors listed have made a substantial, direct, and intellectual contribution to the work, and approved it for publication.
